# COVID‐19 and Ethnic Inequalities in England and Wales^*^

**DOI:** 10.1111/1475-5890.12228

**Published:** 2020-06-26

**Authors:** Lucinda Platt, Ross Warwick

**Affiliations:** ^1^ London School of Economics and Political Science; ^2^ Institute for Fiscal Studies

**Keywords:** COVID‐19, ethnicity, inequality, mortality, occupational segregation

## Abstract

The economic and public health crisis created by the COVID‐19 pandemic has exposed existing inequalities between ethnic groups in England and Wales, as well as creating new ones. We draw on current mortality and case data, alongside pre‐crisis labour force data, to investigate the relative vulnerability of different ethnic groups to adverse health and economic impacts. After accounting for differences in population structure and regional concentration, we show that most minority groups suffered excess mortality compared with the white British majority group. Differences in underlying health conditions such as diabetes may play a role; so too may occupational exposure to the virus, given the very different labour market profiles of ethnic groups. Distinctive patterns of occupational concentration also highlight the vulnerability of some groups to the economic consequences of social distancing measures, with Bangladeshi and Pakistani men particularly likely to be employed in occupations directly affected by the UK's ‘lockdown’. We show that differences in household structures and inequalities in access to savings mean that a number of minority groups are also less able to weather short‐term shocks to their income. Documenting these immediate consequences of the crisis reveals the potential for inequalities to become entrenched in the longer term.

## I. Introduction

There is a growing body of evidence that deaths from COVID‐19 are not equally distributed across the population, with men, older people and those in more deprived areas more severely affected.^1^ Those with underlying health conditions, such as diabetes, heart disease, dementia and Alzheimer's, are also more at risk if they contract the disease.^2^ The economic effects of the coronavirus crisis have also not been equally distributed, with those subject to loss of employment or reduced hours more likely to be young, female and low earners.^3^ While those in some occupations are able to work from home and others are deemed essential workers who continued to go in to work, some sectors of the economy have been almost entirely shut down.

In this paper, we investigate the extent to which different ethnic groups have been more or less affected by the immediate impact of the COVID‐19 crisis, both in terms of exposure to infection and health risks, including mortality, and in relation to their exposure to economic impacts. Given well‐documented ethnic inequalities in health^4^ and in the labour market,^5^ we might expect unequal consequences of COVID‐19 for ethnic minority groups. Yet, we would not necessarily expect these consequences to be similar across all groups. One of the defining characteristics of the ethnic minority population of the UK is its diversity.^6^ The UK's main ethnic groups differ in their demographic profiles, area of residence, migration history, educational attainment, labour market outcomes (including occupational concentration), household and family structure, savings and resources, and health.^7^

This renders it important to consider potential differential impacts on specific ethnic groups, taking into consideration the characteristics that put them more or less at risk. While the generally youthful profile of most minority groups might be expected to reduce mortality risk from COVID‐19 in those populations as a whole, we explore whether this is the case and estimate disproportionality in deaths adjusted for age. We also take account of the geography of overall fatalities and the residential profile of each ethnic group. Beyond this, we focus in particular on occupational concentration in terms of both occupations that put some groups at greater risk of exposure to COVID‐19 and occupations that place them more at risk of short‐ and long‐term economic pressures. We also consider other relevant risk factors that differ across ethnic groups, including health conditions, family structure, savings levels and self‐employment. Being able to identify the potential role of such factors is crucial for developing informed policy responses and for providing meaningful insight into emerging COVID‐related inequalities.

We concentrate on the six largest minority groups in England and Wales – white other, Indian, Pakistani, Bangladeshi, black African and black Caribbean – and compare their mortality and economic vulnerability with those of the white British majority. Our analysis focuses on a limited but crucial set of risk factors that are relevant to both infection risk and economic vulnerability in the short term. To understand the full extent to which different ethnic groups fared relatively better or worse during this crisis will, however, require a longer‐term perspective, encompassing a more comprehensive understanding of infection, morbidity and mortality, as well as evaluating realised economic impacts and the extent of recovery.

## II. Data

We draw on multiple sources of data. For the analysis of vulnerability to infection risks, we use data published weekly by NHS England on registered hospital deaths as well as daily data on confirmed cases of COVID‐19 infections from Public Health England and Public Health Wales. We combine these with data published weekly by the Office for National Statistics (ONS) on all deaths (including those in care homes and elsewhere in the community) in which COVID‐19 was mentioned on the death certificate.^8^ Since these deaths cover England and Wales only, and given that the coding of ethnic group differs in Scotland and Northern Ireland from that used in England and Wales, we focus our analysis on England and Wales, which account for 97 per cent of the UK's non‐white minorities. Information on the residential and demographic profile of each ethnic group comes from the ONS 2011 Census of England and Wales.^9^

For the analysis of occupational exposure, health vulnerability and the economic consequences of COVID‐19, we draw on the most recent data from the UK Labour Force Survey. To enable sufficient sample sizes for individual ethnic groups for analysis and statistical inference, we pool all quarters from the last four years, i.e. quarter 1 2016 to quarter 4 2019.^10^ The Quarterly Labour Force Survey is a survey of adults aged 16 and over living in private households. The survey focuses on issues relating to paid work, such as employment status, occupation, industry, hours and earnings, as well as collecting information on education, training, and family and household composition. For our purposes, importantly it collects information on self‐ascribed ethnic group, using the standard ONS ethnic group categories for England and Wales, as well as country of birth. All data are weighted to be representative of the population as a whole. For evidence of access to savings, we draw on the Wealth and Assets Survey wave 5,^11^ part of a longitudinal study that provides information on the financial and non‐financial assets of individuals and households. Wave 5 collects self‐ascribed ethnic group according to the same ONS categorisation. From both data sets, we report our results graphically but only discuss differences that are statistically significant at conventional levels.

## III. Vulnerability to infection risks

**FIGURE 1:**
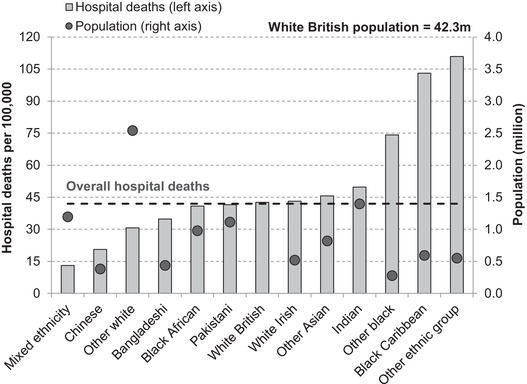
Total registered hospital deaths from COVID‐19 per 100,000 in England by ethnic group *Note*: In 9 per cent of cases, an ethnic group could not be identified; these are excluded. The ‘other white’ population includes the Gypsy and Irish Traveller group. The ‘other ethnic group’ includes the Arab group. *Source*: Authors’ calculations using population data from 2011 ONS Census of England & Wales and NHS England COVID‐19 hospital death figures by ethnicity as of 19 May 2020.

Concerns that ethnic minority groups in the UK are being disproportionately harmed by the spread of COVID‐19 were first triggered by findings from the Intensive Care National Audit and Research Centre (2020) indicating that the shares of critically ill patients from black, Asian and other ethnic groups were greater than their respective population shares. Since then, data published by NHS England on registered hospital deaths by ethnic group have confirmed stark inequalities between ethnic groups, as shown in Figure 1. These data also show the importance of distinguishing between different ethnic groups, rather than considering all non‐white groups as one, which can obscure significant and informative differences.

The white British ethnic group, which accounts for almost 80 per cent of the population of England, has recorded comparable numbers of hospital deaths from COVID‐19 per capita to the Pakistani and black African groups, while the Chinese and mixed ethnicity groups have recorded far fewer hospital deaths per capita. However, among the black Caribbean and ‘other’ (which includes the Arab population) groups, per‐capita hospital deaths are substantially higher than those of the white British majority, and the ‘other black’ group has also recorded a disproportionate number of hospital deaths. While a word of caution may be warranted on the precision of these per‐capita numbers – there is some evidence that the ‘other ethnic group’ population has grown significantly since 2011, for instance^12^ – it is nonetheless clear that there is a lot of variation in hospital deaths between England's ethnic groups.

The unequal effects of the COVID‐19 crisis across ethnic groups are likely to be the result of a complex set of economic, social and health‐related factors. Understanding the role of each of these will require a better understanding of the virus itself, more data than are currently available and additional research. However, there are important differences between the characteristics of the UK's main ethnic groups – in terms of their geography, age, overall health and occupational exposure – that are relevant for understanding why inequalities in vulnerability to infection may arise and for understanding the degree of disproportionality in health outcomes, including mortality. These include substantial differences between the country's main minority ethnic groups, and thus it is important not simply to contrast ethnic minorities as a whole with the white British majority, and not only to compare population shares of all minority or immigrant groups with shares of deaths.

### 1 People and place

**FIGURE 2:**
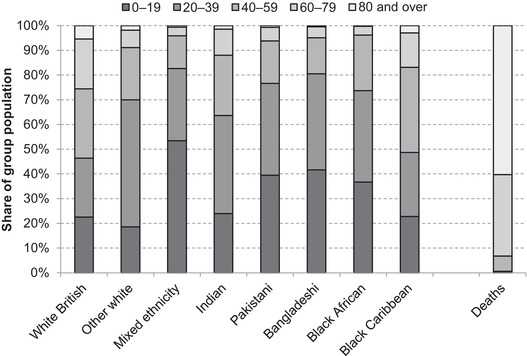
Age distributions of selected ethnic groups in England and Wales and share of overall COVID‐19 deaths by age band *Note*: COVID‐19 deaths are from all places of occurrence (hospital and non‐hospital) in England and Wales. *Source*: Population data from 2011 ONS Census of England & Wales and COVID‐19 death data from ONS weekly occurrences up to 15 May 2020.

Compared with the white British majority, most minority ethnic groups are on average younger (Figure 2). Around a quarter of the white British population in England and Wales are over 60 years of age, compared with 17 per cent of the black Caribbean population, 12 per cent of Indians, and just 6 per cent of Pakistanis and 4 per cent of black Africans. Given the striking concentration of COVID‐19 deaths in older age brackets – with fewer than 10 per cent of COVID‐19 deaths in England and Wales occurring among those aged under 60 (as shown in the rightmost bar of Figure 2) – on their own, such age profiles would be expected to reduce the vulnerability of most minority ethnic groups to the virus relative to the older white British population. However, there are also substantial differences in the age profiles of different minority groups. The black Caribbean ethnic group – which has suffered a notably high number of hospital deaths per capita – has a comparable age profile to the white British majority, whereas those of mixed ethnicity are overwhelmingly young, which may go a long way in explaining the comparatively few numbers of deaths in this category so far.

**FIGURE 3:**
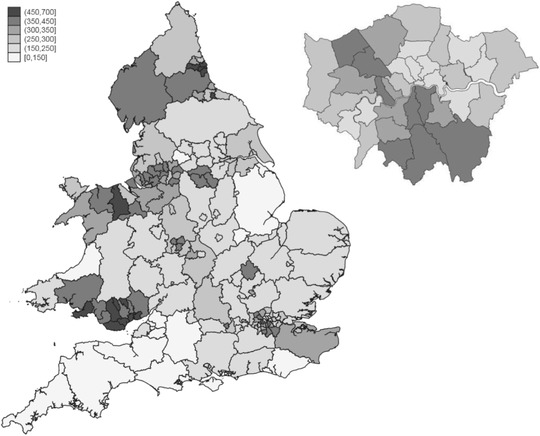
Confirmed COVID‐19 cases per 100,000 population in England and Wales (London boroughs inset) *Note*: Includes only confirmed cases of COVID‐19 in hospitals where the residence of the infected patient has been identified. *Source*: Authors’ calculations based on ONS 2018 mid‐year population estimates and lab‐confirmed COVID‐19 case data from Public Health England and Public Health Wales up to 24 May 2020.

COVID‐19 cases have not been evenly distributed across the country. Therefore, the geographic distribution of ethnic groups is likely to be important in explaining between‐group inequalities in COVID‐19 exposure and health outcomes. The connectivity and population density of Britain's major urban centres have made people in these parts of the country particularly vulnerable to the spread of the virus, as shown in Figure 3. This was especially true in the early stages of the crisis: Birmingham, for instance, was a particular ‘hotspot’ for transmission, and London accounted for close to a third of confirmed cases in England by the end of March. Minority ethnic groups are disproportionately likely to reside in urban areas such as these, making them particularly likely to be exposed to the virus itself. In England and Wales, 60 per cent of the overall black population and 50 per cent of the Bangladeshi population live in London. This is in contrast to 8 per cent of the white British majority, while 13 per cent of the total Pakistani population live in the local authority of Birmingham.

**FIGURE 4:**
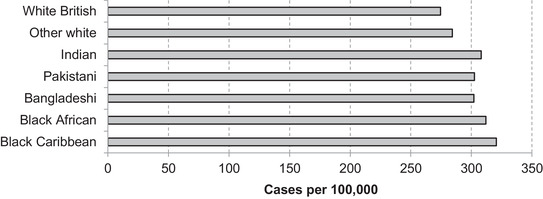
Predicted number of lab‐confirmed COVID‐19 cases per 100,000 of ethnic group, based on local authority of residence in England and Wales *Note*: Predicted number of lab‐confirmed COVID‐19 cases per 100,000 of group population based on geographic distribution of populations and confirmed cases at upper‐tier local authority level. *Source*: Authors’ calculations based on ONS 2011 Census of England & Wales and lab‐confirmed COVID‐19 case data from Public Health England and Public Health Wales up to 24 May 2020.

A more complete mapping of the geographic distribution of ethnic group populations to where cases have been reported so far confirms that minority groups typically reside in parts of the country where more cases have been confirmed (Figure 4). Black Caribbean individuals on average reside in areas with 17 per cent more confirmed cases per capita than white British individuals, for instance.^13^ There are of course important caveats to these case data, which certainly do not paint a complete nor an unbiased picture of the true spread of cases, but nonetheless the implications of the patterns for minority ethnic groups are clear.

**FIGURE 5:**
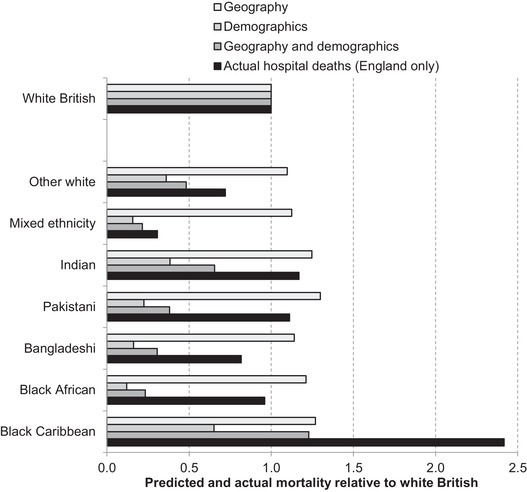
Predicted COVID‐19 fatalities based on geography and demographics, and actual hospital deaths, relative to white British, by ethnic group *Note*: Predictions based on demographics and geography are for COVID‐19 fatalities in England and Wales in all places of occurrence (hospital and non‐hospital deaths). *Source*: Authors’ calculations using COVID‐19 hospital death statistics from Public Health England as of 19 May 2020, COVID‐19 death data from ONS weekly occurrences up to 15 May 2020, and 2011 ONS Census of England & Wales.

Two important factors – age and geography – appear, then, to push in opposite directions in terms of the vulnerability of most minority ethnic groups to infection from COVID‐19. Figure 5 provides a quantification of how the age, sex and geography of ethnic groups might be expected to affect their relative mortality risk from COVID‐19. Using the breakdown of age, sex and region of residence of fatalities from the ONS, alongside these same characteristics of each ethnic group as recorded in the 2011 census, it is possible to predict the number of deaths by ethnic group if these factors were the only relevant determinants. While the ethnicity of fatalities is not available in the ONS data, which include deaths outside of hospitals, comparisons of these projections with actual hospital deaths by ethnicity can provide an indication as to which ethnic groups are suffering the most excess fatalities – that is, the extent to which the number of recorded fatalities looks disproportionate for each group given their population size, age and sex distribution, and location. Because of aggregate differences arising from both coverage and reporting processes and periods, actual and predicted deaths are normalised against the white British group within each data set to facilitate comparisons.

If only location of residence mattered, the white British majority would be expected to have the lowest number of deaths per capita and the Pakistani group the highest. Conversely, if demographics were the only factor, white British individuals would be expected to have the highest mortality, due to the older age profile of that group. Combining both demographics and geography gives a varied picture across groups. On the basis of these factors, one would expect a higher number of fatalities, compared with the white British majority, for black Caribbeans, but lower rates for all other minority ethnic groups.

In reality, the available information on hospital deaths by ethnic group suggests higher per‐capita mortality for all ethnic minorities in Figure 5 than can be explained by demographics and geography alone, as can be seen by comparing the third and fourth bars for each group. The ratio of these two provides an estimate of excess mortality beyond what can be explained by demographics and geography under the assumption that per‐capita hospital deaths for each ethnic group in England are representative of overall per‐capita COVID‐19 fatalities in England and Wales. This ratio varies substantially across ethnic groups, from 4.1 for black Africans, to 2.9 for Pakistanis, 2.0 for black Caribbeans and 1.8 for Indians. For the white Irish group (not shown on Figure 5), the ratio is 0.5, suggesting disproportionately few fatalities among these people given their age profile and where they live.

**FIGURE 6:**
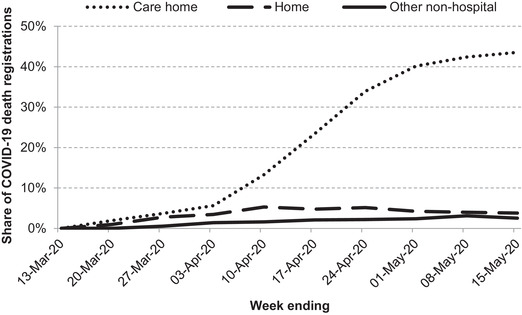
Share of weekly registered COVID‐19 deaths occurring outside of hospitals in England and Wales *Note*: ‘Other non‐hospital’ includes all other places where deaths may occur, including hospices, prisons, hotels and other people's homes. *Source*: COVID‐19 death data from ONS weekly registrations up to 15 May 2020.

However, it is likely that non‐hospital deaths, for which a breakdown by ethnicity is not available, have implications for overall inequalities in mortality. Such fatalities accounted for an increasing proportion of total deaths as the crisis unfolded, with the majority of non‐hospital deaths occurring in care homes (Figure 6). Of COVID‐19 death registrations in England and Wales by 15 May 2020, care home deaths accounted for 28 per cent, while deaths at home accounted for 5 per cent.

**FIGURE 7:**
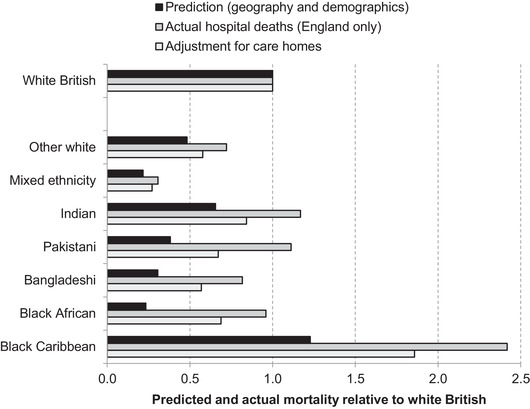
Accounting for care home deaths in relative mortality of ethnic groups *Note*: Adjustment assumes that all care home deaths in each ethnic group are proportionate to ethnic group populations in care homes in England and Wales, and that hospital deaths per capita in each group are the same in Wales as in England. *Source*: Authors’ calculations using COVID‐19 hospital death statistics from Public Health England as of 19 May 2020, COVID‐19 death data from ONS weekly occurrences up to 15 May 2020, and 2011 ONS Census of England & Wales.

In 2011, 94 per cent of the care home population were white British, and 97 per cent were in a white ethnic group overall. Thus, it is likely that compared with deaths occurring in hospitals, disproportionately more care home deaths will come from white ethnic groups. However, as shown in Figure 7, such deaths are not able to account for the disproportionalities in hospital deaths (given geography, age and sex) between minority ethnic groups compared with the white British majority. The graph shows the same predicted (based on geography and demographics) and actual hospital mortality results as in Figure 5, alongside an additional bar showing how registered COVID‐19 deaths in care homes would affect the latter numbers if these deaths occurred in proportion to the ethnic make‐up of care homes. While estimated excess fatalities (the ratio of the first and third bars for each group) are reduced, the gap remains large for most ethnic minority groups. The ratio of actual to expected mortalities is reduced to 2.9 for black Africans, 1.8 for Pakistanis,1.5 for black Caribbeans and 1.3 for Indians, for instance.

**FIGURE 8:**
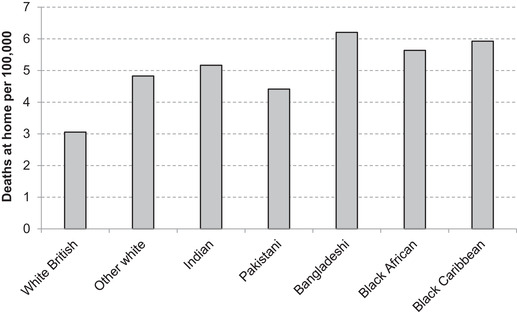
Predicted deaths at home per 100,000 individuals according to local authority of residence in England and Wales, by ethnic group *Note*: Shows the number of deaths at home overall in the local authority of residence of a member of each of the ethnic groups on average – the ethnicity of those who have died at home is not currently available. *Source*: Authors’ calculations using COVID‐19 death data from ONS weekly occurrences up to 15 May 2020 and 2011 ONS Census of England & Wales.

Understanding how other COVID‐19 fatalities, including deaths at home, may vary across ethnic groups is challenging with the data that are currently available. However, the geographic distribution of deaths at home suggests they are disproportionately happening in areas with large populations of ethnic minorities (Figure 8). This may suggest that deaths at home go in the other direction to deaths in care homes in terms of their effect on overall ethnic inequalities in COVID‐19 deaths. It is not possible, however, to draw strong conclusions on the basis of this evidence, since it may be the case that a white British person is more likely than an ethnic minority person to die at home rather than in a hospital in a given area.

It is crucial to note that the ONS data suggest that, based on trends observed in previous years, there have also been a large number of additional ‘excess deaths’ that have not been officially attributed to COVID‐19 but are likely to be the direct or indirect result of the virus. In eight weeks up to 15 May 2020, such deaths equated to 24 per cent of the official total attributed directly to COVID‐19, and an increasing proportion took place in care homes. We lack detailed information about these deaths at the time of writing – who is dying, and why and where exactly. The consequences of these deaths for ethnic inequalities will become clearer over time.

### 2 Occupational risks

While geography and demographics clearly have a role to play, and can reconcile some differences across ethnic groups, fatalities from COVID‐19 among many minority ethnic groups are clearly much higher than would be expected given their age, sex and location. Other ongoing research has reached similar conclusions either using publicly available data on deaths^14^ (as in this paper) or using individual data from hospital records.^15^ There are, of course, a range of other factors that could be at play. The risk of transmission may vary for different individuals and groups within the same community, and the nature of people's jobs – or the occupation of the people they live with – is likely to be an important factor for their risk of infection. While many workers were furloughed, lost their jobs or worked from home, ‘key workers’ faced continuing risks from contact with contagious individuals. While the current evidence does not allow us to unpick the relative importance of occupational risk and other correlated factors, the different labour market profiles of ethnic groups appear crucial to consider in terms of exposure to the virus.

**FIGURE 9:**
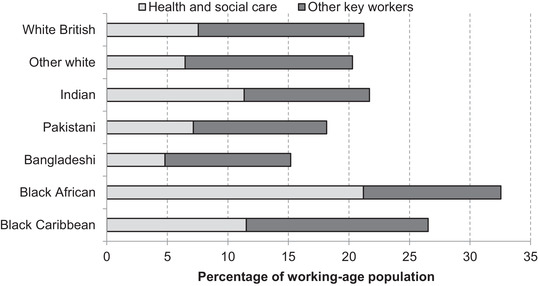
Share of key workers among those of working age in each of seven ethnic groups *Note*: Key workers are identified based on government guidance from 19 March 2020 using four‐digit Standard Occupational Classification (SOC) codes to identify key worker jobs in health and social care, education, public services, food, public order and transport. For further details, see Farquharson, Rasul and Sibieta (2020). Shares represent the proportion of the working‐age population (aged 16–64) (excluding students) of each group that are in the identified occupations. *Source*: Quarterly Labour Force Survey, quarter 1 2016 to quarter 4 2019.

Figure 9 shows the shares of working‐age populations across seven selected ethnic groups who are key workers and, among those, the shares who are health and care workers, since the latter may be particularly likely to risk contact with those infected with the virus. Some – but not all – minority ethnic groups may well face greater infection risks because of the types of work that they do. Black Africans and black Caribbeans are over‐represented among key workers overall. This is particularly striking for the black African ethnic group, where almost a third of the working‐age population are employed in key worker roles and one in five in health and social care jobs specifically. This translates to a working‐age black African being 50 per cent more likely to be a key worker than a white British working‐age person, and nearly three times as likely to be a health and social care worker.

For some groups who are highly concentrated in particular healthcare occupations, it is possible to look in even more detail. For example, those of Indian ethnicity make up only 3.2 per cent of the working‐age population, but over 14 per cent of doctors. While 37 per cent of the UK's doctors are foreign‐born (despite the fact that only 18 per cent of the working‐age population are foreign‐born), nearly one in ten are from India. Black Africans, meanwhile, make up a smaller share (2.2 per cent) of the working‐age population but account for 7 per cent of nurses. Nurses accounted for the largest share of deaths among NHS staff in a recent analysis and a majority of them were from minority groups.^16^

**FIGURE 10:**
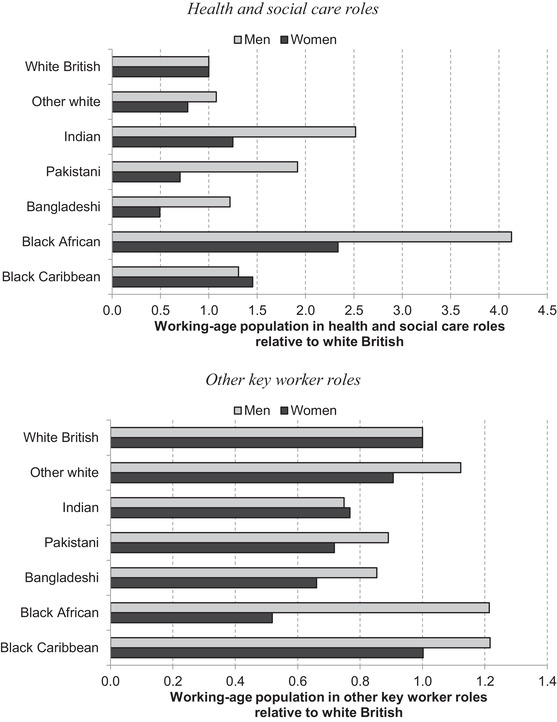
Share of key workers in each of seven ethnic groups relative to white British, by sex *Note*: See note for Figure 9. *Source*: Quarterly Labour Force Survey, quarter 1 2016 to quarter 4 2019.

Given the mounting evidence that men are particularly vulnerable to the virus, it is also important to understand whether there are further differences when broken down by sex. Figure 10 reveals substantial differences between men and women in the prevalence of key workers. Overall, women are much more likely to work in a key occupation, and particularly in health and social care roles; Figure 10 normalises against the white British ethnic group by sex in order to compare differences between groups. In terms of differential vulnerability to infection between ethnic groups, it is notable that compared with white British men, in some cases minority ethnic men are relatively more likely to work in other key occupations (bottom panel of Figure 10), while this is not the case for women in any minority group. The higher relative chances of minority group men working in a key worker occupation are particularly marked for health and social care workers (top panel of Figure 10). For instance, Indian and black African men are 150 per cent and 310 per cent respectively more likely to work in health and social care than white British men. In contrast, Indian women are 25 per cent more likely and black African women 130 per cent more likely than white British women to work in these roles. This implies the sex‐adjusted occupational risk is likely to be higher for these minority groups.

Looking beyond these groups of occupations, ONS analysis highlighted substantial differences between individual occupations in age‐adjusted mortality from COVID‐19.^17^ Specifically, it noted excess age‐adjusted mortality for both men and women social care workers (though not for healthcare workers), and for men working in some other key worker roles such as security personnel. Even though exposure to the virus may have been reduced by the lockdown, those men with jobs in public‐facing roles such as sales and retail assistants, and bus and coach drivers, also had higher mortality risks, as did chefs, who often operate in confined settings. These are all occupations in which minority groups are over‐represented. For example, black African men work in social care roles at seven times the rate of white British men, and black African women are four times as likely as their white British counterparts to do so. Nearly 6 per cent of black African men of working age work as security guards, alongside 3 per cent of black Caribbean and Pakistani men, with minorities together accounting for 40 per cent of all security guards despite making up less than a quarter of the working‐age population. Meanwhile, 8 per cent of Bangladeshi men are chefs. Further analysis of occupation‐specific death rates will therefore shed more light on the drivers of the overall ethnic differences in mortality we observe.^18^

### 3 Other relevant factors

Research from the ONS showed a clear gradient in age‐standardised mortality from COVID‐19 according to local deprivation, with the most deprived parts of England and Wales suffering deaths at more than twice the rate of the least deprived areas.^19^ Although the excess fatality estimates in this paper account for geography at the local authority level, deprivation can vary substantially within a local authority and is correlated with ethnic composition. However, further ONS research considered ethnic differences in COVID‐19 mortality up to 10 April by linking death registrations with census records.^20^ This found that neither local deprivation (as measured by decile of the Index of Multiple Deprivation according to residence) nor proxies for socio‐economic status could explain much of the difference between ethnic groups.

Apart from infection risk at work, some ethnic groups may be more at risk of community transmission due to different family and household structures. South Asian ethnic groups are much more likely to live in larger households, for instance, which all else equal will make transmission more likely. Taking London as an example, just under a third of households are a single person, but among households where the household head is Bangladeshi, Indian and Pakistani, the figures are 11 per cent, 17 per cent and 13 per cent, respectively.

Related to this, compared with white British households, minority ethnic groups also tend to be more likely to live in overcrowded accommodation – even after controlling for region of the country. Fewer than 2 per cent of white British households in London have more residents than rooms; in contrast, this figure is nearly 30 per cent for Bangladeshi households, 18 per cent for Pakistani households and 16 per cent for black African households. Such conditions are likely to make self‐isolation much more difficult and to increase opportunities for within‐household transmission for some ethnic groups. However, such overcrowding is not so prevalent for black Caribbeans, who nevertheless face the highest number of hospital deaths per capita thus far, while Bangladeshi death rates are lower.

There are also notable inequalities in underlying health conditions and physical health that are likely to be crucial. Data on COVID‐19 deaths in hospitals from NHS England show a striking concentration of fatalities among individuals with pre‐existing health conditions. As of 19 May, 95 per cent of all deaths had at least one attributable pre‐existing health condition. More than a quarter of deaths were of individuals with diabetes, and the prevalence of kidney disease, dementia, and respiratory and circulatory problems is also high.

Being overweight or obese has been identified as a potential risk factor, and 74 per cent of England's adult black population is overweight or obese – 10 percentage points more than for the white British population and 17 percentage points more than for the Asian population overall.^21^ Black and south Asian ethnic groups have been found to have much higher rates of diabetes than the population as a whole, and older Pakistani men have been found to have particularly high levels of cardiovascular disease.^22^

**FIGURE 11:**
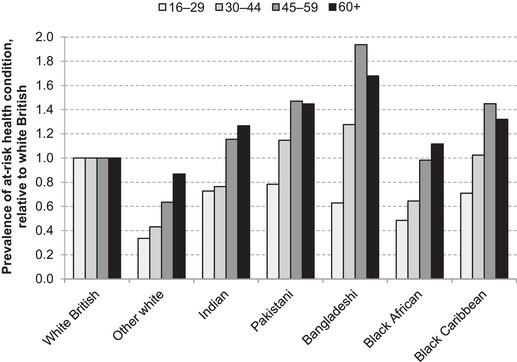
Rates of long‐term health conditions comprising risks for COVID‐19, by ethnic group and age in England and Wales, relative to white British *Note*: Self‐reported long‐term health problems, where ‘at risk’ includes one or more of chest and breathing problems, heart, blood pressure or circulation problems, and diabetes. *Source*: Quarterly Labour Force Survey, quarter 1 2016 to quarter 4 2019.

Figure 11 shows the proportion of individuals from different ethnic groups in each age band who report having a long‐term, ‘at‐risk’ health problem. Particularly in older age brackets, Indian, Pakistani, Bangladeshi and black Caribbean individuals are much more likely than white British people to report one or more of these health problems that are likely to increase their mortality risk from COVID‐19. It may well be that underlying health conditions such as these can explain part of the disproportionality in hospital death figures across ethnic groups so far.

In sum, there is clear evidence for disproportionality in COVID‐19 mortalities for a number of ethnic groups, after accounting for their age profiles and places of residence. While it is difficult to say definitively with the data that are currently available, the clustering of some minority groups in key worker occupations (and in health and social care roles in particular), and greater susceptibility to relevant long‐term conditions, are likely to be contributing factors to the observed disparities.

The consequences of COVID‐19 for health will also find expression in the longer term through economic impacts.^23^ The next section considers which groups are more or less likely to have suffered from the recent lockdown.

## IV. Economic vulnerability

In order to confront the public health crisis, in March 2020 the UK government implemented unprecedented social distancing measures. These created a unique type of economic crisis, the effects of which are likely to be experienced unequally as different sectors and household types are more or less exposed to the effects of the restrictions put in place. In this section, we consider how the economic characteristics of the main ethnic groups in England and Wales may result in different risks from the short‐run effects of the crisis. We focus on those of working age given they are potentially most at risk from the economic crisis. This covers larger shares of some groups than of others given differing age profiles (see Figure 2). In the medium and long term, additional unequal impacts are likely to arise through disruption to education and occupational and geographical mobility, and from policy responses to the crisis. These are crucial to understand but are beyond the scope of this paper.

### 1 Family characteristics

As well as substantial differences in age profiles, the main ethnic groups also have very different characteristics in terms of household structure and labour market participation, both of which are important for understanding how changes in individual employment and earnings may affect overall between‐group inequalities. Fewer Pakistani and Bangladeshi individuals are in paid work than for other groups, largely owing to lower labour market participation amongst women. This might suggest that, on aggregate, these groups are less exposed to changes in economic circumstances resulting from the crisis; however, alongside black Africans and black Caribbeans, they are especially likely to reside in families where only one person is in paid work (Table 1), implying greater household‐level exposure.

**TABLE 1:** Economic activity of working‐age populations by ethnic group

*Group*	*In paid work*	*Number in paid work in family*		
		0	1	2+
White British	79.7%	11.6%	30.0%	58.4%
Other white	85.1%	5.2%	39.7%	55.1%
Indian	80.1%	6.1%	35.3%	58.6%
Pakistani	61.9%	12.9%	47.8%	39.3%
Bangladeshi	60.3%	12.4%	49.3%	38.3%
Black African	79.7%	14.3%	45.2%	40.5%
Black Caribbean	85.1%	16.5%	42.5%	41.0%

*Note*: ‘In paid work’ is the sum of the employed and self‐employed. Estimates among those of working age (aged 16–64) excluding students.

*Source*: Quarterly Labour Force Survey, quarter 1 2016 to quarter 4 2019.

Table 2 shows that black Africans, Bangladeshis and Pakistanis are also more likely to have dependent children, implying that income shocks to the working‐age population of these groups are more likely to have consequences for children. The shares of black Africans and black Caribbeans who are lone parents with dependent children are particularly high, and such groups may well be especially vulnerable to any loss of income, due to absence of a within‐household buffer. In the context of disruption to schools and childcare facilities, even without loss of work, lone‐parent families may struggle to balance work and care. By contrast, couple families with dependent children with just one worker may be better placed to manage family arrangements and maintain employment (though this remains fraught with difficulties^24^).

**TABLE 2:** Family structure by ethnic group

*Group*	*Single‐person family: no dependent children*	*Couple: no dependent children*	*Lone parent: with dependent children*	*Couple: with dependent children*	*Average number of children under 16*
White British	18.1%	40.6%	10.0%	31.4%	0.6
Other white	16.8%	32.3%	8.0%	42.9%	0.7
Indian	12.8%	32.9%	4.7%	49.7%	0.8
Pakistani	10.1%	21.4%	11.4%	57.1%	1.3
Bangladeshi	8.8%	16.5%	6.8%	67.9%	1.4
Black African	20.6%	14.4%	30.4%	34.5%	1.2
Black Caribbean	36.9%	17.5%	28.2%	17.4%	0.6

*Note*: Those of working age (aged 16–64) excluding students. First column is the sum of single‐person families with no children and lone parents with no dependent children. Second column is the sum of couples with no children and couples with no dependent children.

*Source*: Quarterly Labour Force Survey, quarter 1 2016 to quarter 4 2019.

### 2 Employment in shut‐down sectors

Section III.2 showed clear differences between ethnic groups in their likelihood of being employed in key worker roles – positions of heightened importance during the crisis. At the other end of the spectrum, the government‐imposed lockdown brought the majority of economic activity to a halt in sectors such as hospitality, leisure and transport. Joyce and Xu (2020) indicate that this lockdown directly affected 15 per cent of employees but that the proportion varied significantly by age group, by sex and across the earnings distribution. This section uses the same approach to identifying employment in sectors directly affected by the lockdown to consider how the labour market profiles of ethnic groups in England and Wales affect their exposure to the measures put in place.^25^

**FIGURE 12:**
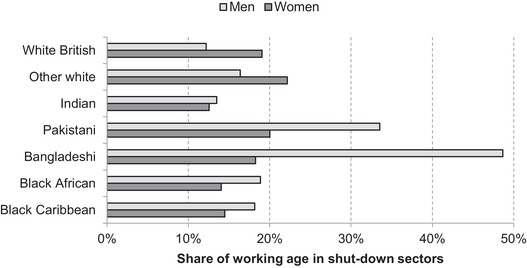
Share of working‐age population in shut‐down sectors in England and Wales, by ethnic group and sex *Note*: Shares represent the percentage of the working‐age population (aged 16–64) (excluding students) of each group in shut‐down sectors. *Source*: Quarterly Labour Force Survey, quarter 1 2016 to quarter 4 2019.

While differences in family structures imply that the effects of the crisis would vary across groups even if members of each ethnic group were equally likely to work in shut‐down sectors, the chances of working in a directly affected industry are not evenly distributed. Figure 12 shows substantial differences in the share of each ethnic group working in shut‐down sectors. The number of individuals working in these sectors is displayed as a percentage of the whole working‐age population of the group, rather than as the share of those economically active or employed, in order to measure the effect on the group as a whole and to ensure estimates are not affected by differences in labour market participation – particularly among women.

While in the population as a whole women are more likely to work in sectors affected by the lockdown, this is driven by the white ethnic groups. In contrast, across many minority ethnic groups, men are more likely to work in shut‐down sectors than women. This is particularly striking for the Bangladeshi and Pakistani groups, with men from the former four times as likely to work in shut‐down sectors as white British men, due in large part to their concentration in the restaurant sector, and the latter nearly three times as likely, due in part to their concentration in taxi driving. Working‐age women from these two minority groups are no more likely to work in shut‐down sectors than white British women. Black African and black Caribbean men are both 50 per cent more likely than white British men to work in shut‐down sectors.

**FIGURE 13:**
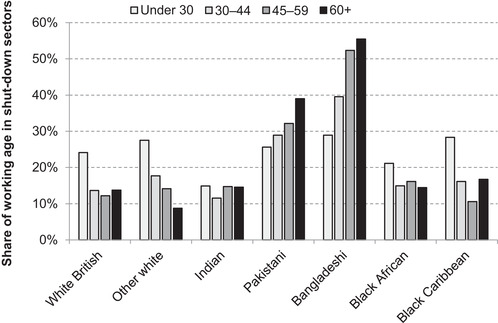
Share of working‐age population in shut‐down sectors in England and Wales, by ethnic group and age *Note*: Shares represent the percentage of the working‐age population (aged 16–64) (excluding students) of each group in shut‐down sectors. *Source*: Quarterly Labour Force Survey, quarter 1 2016 to quarter 4 2019.

Many minority groups have younger age profiles than the white British group (see Figure 2), and younger workers are also more likely to work in shut‐down sectors.^26^ Thus, Figure 13 shows the share of each ethnic group that works in a shut‐down sector by age band. Within each age band, the between‐group differences are indeed reduced, with only small differences among those aged under 30. However, the greater shares of Pakistani and Bangladeshi groups exposed to the lockdown are not driven by their age profiles. They disproportionately work in directly affected industries in older age brackets, with the rates increasing with age in contrast to the pattern for the population overall. It is particularly striking that over half of Bangladeshis aged between 45 and 59 are employed in shut‐down sectors. This compares with just 12 per cent of white British in the same age group.

### 3 Family circumstances of workers in shut‐down sectors

**FIGURE 14:**
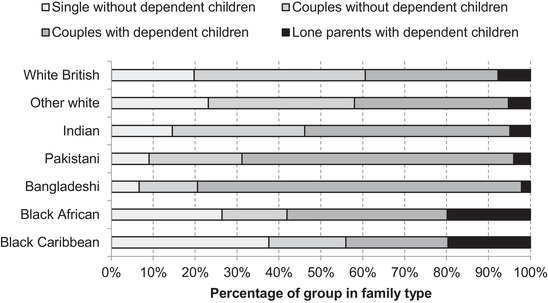
Family status of those employed in shut‐down sectors in England and Wales, by ethnic group *Note*: Family types as for Table 2. *Source*: Quarterly Labour Force Survey, quarter 1 2016 to quarter 4 2019.

Among those affected, the likelihood that they will be protected by the income of other household members varies. Joyce and Xu (2020) found that, in general, household incomes provide something of a buffer for those facing income losses due to working in shut‐down industries. However, Table 1 shows that, overall, Pakistanis, Bangladeshis, black Africans and black Caribbeans are less likely to be living in households with two or more earners. Additionally, the extent to which children are impacted by parents’ loss of work will vary by the extent to which affected workers are parents. Figure 14 shows family type by ethnic group of those in shut‐down industries. While affected workers from all minority groups except black Caribbeans are more likely to be living as couples with dependent children than the white British group, black Africans and black Caribbeans are more likely to be living in lone‐parent families.

**FIGURE 15:**
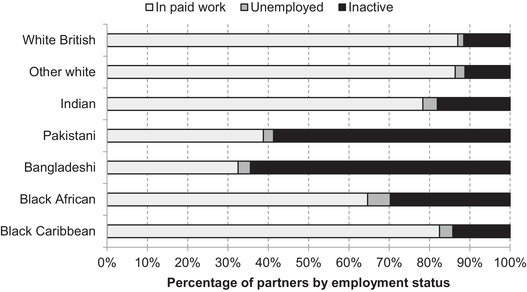
Employment status of partners of those employed in shut‐down sectors in England and Wales, by ethnic group of shut‐down sector worker *Note*: Base is those of working age employed in shut‐down sectors and who are living in a couple. Ethnic group is that of the person working in the shut‐down sector. *Source*: Quarterly Labour Force Survey, quarter 1 2016 to quarter 4 2019.

Focusing just on those living in couples, Figure 15 shows the employment status of partners of those working in shut‐down sectors. Among two‐person households, there are again substantial variations in the economic status of partners: while white British and other white populations are very likely to have an employed or self‐employed partner, this is not the case for Pakistani and Bangladeshi workers.

**FIGURE 16:**
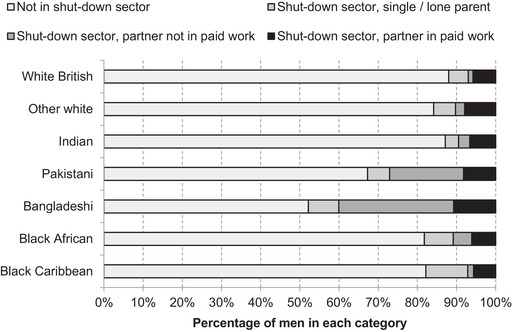
Working in a shut‐down sector and partner's economic status, by ethnic group of shut‐down sector worker, men only *Note*: Sample is working‐age (16‐ to 64‐year‐old) men in each ethnic group. *Source*: Quarterly Labour Force Survey, quarter 1 2016 to quarter 4 2019.

This follows from the fact that Pakistanis and Bangladeshis in shut‐down sectors are more likely to be men, and women from these groups are less likely to be in paid employment. The combination of varied occupational exposure to the lockdown at different life stages and for men and women, combined with different rates of women's employment across groups, means that 29 per cent of Bangladeshi men both work in a shut‐down sector and have a partner who is not in paid work and therefore is not able to provide an income buffer (Figure 16). This is the case for only 1 per cent of white British men.

**FIGURE 17:**
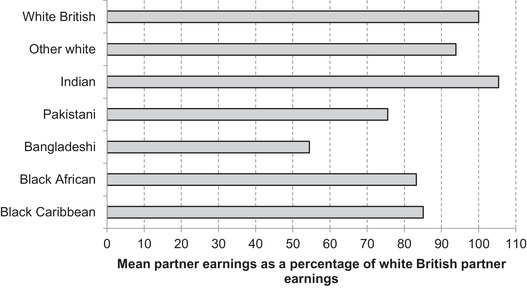
Relative earnings of employed partners of those in shut‐down sectors in England and Wales, by ethnic group of shut‐down sector worker *Note*: Sample is those of working age (aged 16–64) employed in shut‐down sectors, who are living in a couple and whose partner is in work. Relative earnings based on gross weekly earnings. *Source*: Quarterly Labour Force Survey, quarter 1 2016 to quarter 4 2019, waves 1 and 5 only.

Moreover, even for those with partners in paid work, there are substantial differences in average weekly earnings, as Figure 17 shows. Partner earnings for Pakistanis, Bangladeshis, black Africans and black Caribbeans in shut‐down industries are all lower than those for the white British majority.

### 4 Self‐employment and income risks

The Coronavirus Job Retention Scheme (CJRS) has provided income support for many employed in shut‐down sectors and in other sectors, reducing the potential income loss in the short term at least. For self‐employed individuals, the Self‐Employment Income Support Scheme (SEISS) has provided generous support on average, but applicants faced a wait until late May to receive funds and some of the self‐employed were not eligible or received amounts much less than their recent earnings. Blundell and Machin (2020) have shown that those who are self‐employed have been particularly severely affected by the crisis.

**FIGURE 18:**
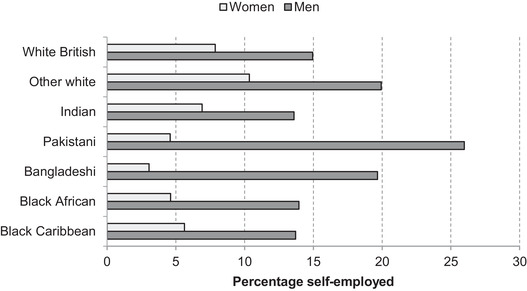
Share of working age in self‐employment in England and Wales, by ethnic group and sex *Note*: Share of those of working age (aged 16–64) in each group excluding students. *Source*: Quarterly Labour Force Survey, quarter 1 2016 to quarter 4 2019.

Among those of working age, as Figure 18 shows, Pakistani and Bangladeshi men are much more likely to be in self‐employment than the overall population, meaning these groups are likely to be particularly hard hit. These are also among the groups who are less likely to have additional earners at home and among those more likely to have dependent children.

**FIGURE 19:**
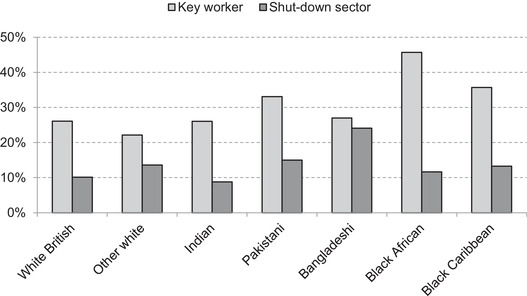
Share of employment earnings from key worker roles and shut‐down sectors in England and Wales, by ethnic group *Note*: Sum of earnings from employment (self‐employment earnings are not reported) by ethnic group at the individual level. In 3 per cent of cases, key workers and shut‐down sectors overlap. *Source*: Quarterly Labour Force Survey, quarter 1 2016 to quarter 4 2019, waves 1 and 5 only.

Figure 19 presents an overall quantification of the direct individual earnings exposure of each ethnic group to the lockdown, as well as the share of employment earnings that comes from key worker occupations for comparison. In aggregate, the Bangladeshi ethnic group appears to be most directly economically affected by the lockdown, with a quarter of employment earnings received from shut‐down sectors in that group. The black African group provides an interesting contrast, where the extremely high share in key worker roles – and in health and social care in particular – renders the group less economically vulnerable than other minority ethnic groups, but potentially at the cost of greater occupational exposure to infection.

**FIGURE 20:**
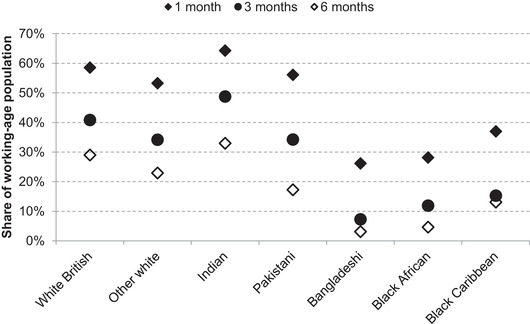
Months of household income that can be covered by liquid financial assets (savings accounts, current accounts, ISAs), by ethnic group *Note*: Working‐age individuals only (aged 20–64). Liquid financial assets are the sum of funds held in current accounts (net of overdraft), savings accounts and ISAs at the household level. Ethnic group is reported individually. *Source*: Authors’ calculations using the Wealth and Assets Survey wave 5 (2014–16).

For many households, even short‐term income shocks can present a serious challenge to their finances. Overall, 60 per cent of working‐age individuals live in households with accessible savings sufficient to cover one month of household income, but this varies substantially by ethnic group, as shown by Figure 20. Among working‐age Bangladeshi, black Caribbean and black African individuals, only around 30 per cent live in households with enough saved in current accounts, savings accounts and ISAs to cover one month of household income, and around 10 per cent can cover three months of income. This latter figure is approximately a fifth of that for the Indian ethnic group, and a quarter of that for the white British majority. For some of those not supported by the CJRS, who either faced the five‐week wait to access universal credit once a successful application had been made or, if self‐employed, had to wait until late May to receive support from the SEISS, the lockdown in the UK will have been particularly economically challenging.

## V. Conclusions

This paper paints a complex picture, with much still unknown about the unequal effects of the COVID‐19 crisis on different ethnic groups in England and Wales in the short term and in the future. Some minority groups have already been disproportionately exposed to risk of infection, and the ‘lockdown’ also has implications for ethnic inequalities. There is no single narrative that can describe or account for the impacts of the current crisis on all minority groups.

Overall, given demographic and geographic profiles, most minority ethnic groups have suffered excess hospital fatalities in England. Official non‐hospital deaths can only account for a small part of the disparity compared with the white British majority so far. Age and location clearly play a role – and seem to explain important differences between different minority groups – but they do not tell the full story. Underlying health conditions, occupational exposure and a range of other factors are likely to be relevant, with some more important for particular groups: middle‐aged and older Bangladeshis have high rates of underlying health problems, and black Africans and Indian men are particularly exposed to the virus due to their prevalence in health and social care roles. The importance of each factor for each group will become clearer as more research is undertaken.

Ethnic groups also vary substantially in their economic vulnerability under the restrictions put in place in March 2020. White other and Indian ethnic groups face lower economic risks and are more comparable to white British in this regard. Bangladeshi and Pakistani groups, by contrast, appear to be particularly at risk due to the high percentage of each group working in shut‐down sectors and/or in self‐employment, combined with the prevalence of single‐earner households which reduces the potential for income buffers within the household. The pervasiveness of key worker employment in other minority groups reduces their risk of income losses, while leaving them at a heightened risk of exposure to the virus itself. Both scenarios, though, are in part a consequence of the way the current labour market draws on both immigrant and ethnic minority workers to fulfil roles in care, transport and delivery sectors and in the more marginal hospitality and self‐employed sectors.

Our findings draw attention to the ways in which minority and immigrant groups tend to be channelled into specific occupational niches, and the implications of that occupational segregation for individual and family vulnerability when a shock such as the current COVID‐19 crisis arises. They beg the question of why and how such occupational structures are maintained, and the role of policy in mitigating the risks associated with critical but often marginalised or poorly valued roles.

## Footnotes

1 Williamson et al., 2020.

2 Office for National Statistics, 2020d.

3 Andrew et al., 2020; Joyce and Xu, 2020.

4 For example, Karlsen and Nazroo (2011).

5 For example, Longhi and Brynin (2017).

6 Modood et al., 1997.

7 Platt, 2019.

8 See further Office for National Statistics (2020a).

9 Office for National Statistics, 2011.

10 Office for National Statistics, 2019a–o and 2020b.

11 Office for National Statistics, 2020g.

12 Office for National Statistics, 2019p.

13 These geographical variations were stronger in the earlier stages of the spread of the virus. As mortality lags infection, the changing geography of cases may yet drive further changes in the ethnic composition of fatalities.

14 For example, Aldridge et al. (2020).

15 Williamson et al., 2020; Holman et al., 2020.

16 Cook, Kursumovic and Lennane, 2020.

17 Office for National Statistics, 2020c.

18 Note that we cannot say whether it is the occupation per se that is driving the occupational disparities in COVID‐19 deaths. It is possible that the over‐representation of ethnic minorities in these jobs might be linked to the occupational death rates via other channels that increase minorities’ risks of mortality.

19 Office for National Statistics, 2020e.

20 Office for National Statistics, 2020f.

21 Cabinet Office, 2020.

22 Health and Social Care Information Centre, 2005.

23 Jones, Davies and Drinkwater, 2018.

24 See Andrew et al. (2020).

25 Specifically, the sectors classed as being directly affected by the lockdown are (with four‐digit Standard Industrial Classification (SIC) codes in parentheses): non‐food, non‐pharmaceutical retail (4719, 4730–4772, 4776–4799); passenger transport (4910, 4931–4939, 5010, 5030, 5110); accommodation and food (5510–5630); travel (7911–7990); childcare (8510, 8891); arts and leisure (9001–9329 except ‘artistic creation’ 9003); personal care (9601–9609 except ‘funeral and related activities’ 9603); and domestic services (9700).

26 Joyce and Xu, 2020.
